# The hospital anxiety and depression rating scale: A cross-sectional study of psychometrics and case finding abilities in general practice

**DOI:** 10.1186/1471-244X-5-46

**Published:** 2005-12-14

**Authors:** Ingrid Olssøn, Arnstein Mykletun, Alv A Dahl

**Affiliations:** 1 Department of Psychiatry, Innlandet Hospital HF, Skolegt 32, 2318 Hamar, Norway; 2 Research Centre for Health Promotion, University of Bergen, 5015 Bergen, Norway; 3 Norwegian Institute of Public Health, Division of Epidemiology, Department of Mental Health, Oslo, Norway; 4 Department of Clinical Cancer Research, Rikshospitalet-Radiumhospitalet Trust, 0310 Oslo, Norway

## Abstract

**Background:**

General practitioners' (GPs) diagnostic skills lead to underidentification of generalized anxiety disorders (GAD) and major depressive episodes (MDE). Supplement of brief questionnaires could improve the diagnostic accuracy of GPs for these common mental disorders.

The aims of this study were to examine the usefulness of The Hospital Anxiety and Depression Rating Scale (HADS) for GPs by: 1) Examining its psychometrics in the GPs' setting; 2) Testing its case-finding properties compared to patient-rated GAD and MDE (DSM-IV); and 3) Comparing its case finding abilities to that of the GPs using Clinical Global Impression-Severity (CGI-S) rating.

**Methods:**

In a cross-sectional survey study 1,781 patients in three consecutive days in September 2001 attended 141 GPs geographically spread in Norway. Sensitivity, specificity, optimal cut off score, and Area under the curve (AUC) for the HADS and the CGI-S were calculated with Generalized Anxiety Questionnaire (GAS-Q) as reference standard for GAD, and Depression Screening Questionnaire (DSQ) for MDE.

**Results:**

The HADS-A had optimal cut off ≥8 (sensitivity 0.89, specificity 0.75), AUC 0.88 and 76% of patients were correctly classified in relation to GAD. The HADS-D had by optimal cut off ≥8 (sensitivity 0.80 and specificity 0.88) AUC 0.93 and 87% of the patients were correctly classified in relation to MDE. Proportions of the total correctly classified at the CGI-S optimal cut-off ≥3 were 83% of patients for GAD and 81% for MDE.

**Conclusion:**

The results indicate that addition of the patients' HADS scores to GPs' information could improve their diagnostic accuracy of GAD and MDE.

## Background

An important task for general practitioners (GPs) is to diagnose and treat depressions and anxiety disorders, which are among the most common and amenable mental disorders in their practice [1]. The literature shows that the GPs' diagnostic skills concerning these common disorders are moderately good [1-8], and somewhat better for major depressive episodes (MDE) than for generalized anxiety disorder (GAD) [8]. A prospective cohort study of depression in primary care, found that the WHO-5 well being index (WHO-5, 5 items) had significantly higher sensitivity than the GPs' clinical diagnosis when the Composite International Diagnostic Interview (CIDI) was used as gold standard [9]. The depression module of the brief patient health questionnaire (B-PHQ, 9 items) had significantly higher specificity than GPs' clinical diagnoses, and GPs' diagnoses had significantly higher specificity than the WHO-5. The sensitivity and specificity of screening instruments for GAD in general practice has hardly been investigated [10,11].

Reviews [12,13] show that the Hospital Anxiety and Depression Rating Scale (HADS) [14] is widely used as a brief self-rating instrument for both dimensional and categorical aspects of anxiety and depression in both epidemiology and specialist care. In these settings the psychometric properties of the HADS are excellent [15,16]. Until now the factor structure, the internal consistency, and the inter-correlation and homogeneity of the HADS sub-scales have not been described in the context of general practice. The case-finding abilities of the HADS in relation to DSM-III/DSM-IV and ICD-10 defined anxiety disorders and depressions by the use of a score ≥ 8 as cut-off are considered good with few false negatives, but a definite proportion of false positives. In clinical practice a positive screening typically results in further evaluation. Considering the brevity and feasibility of the HADS, it should be useful for screening of patients in general practice, but studies of the HADS from that part of the health services are few and inconsistent as to cut-off scores for caseness [17-20]. These points indicate the need for more data on the case-finding abilities of the HADS subscales in general practice.

### Aims of the study

This study from Norwegian general practice has the following aims: 1) To examine the psychometric features of the HADS rated by patients in the primary care setting; 2) To test the case-finding properties of the HADS in relation to the diagnoses of GAD and MDE based on patient-rating of their diagnostic criteria according to DSM-IV as reference standards; and 3) To compare the case finding abilities of the HADS rated by patients to that of GPs using the Clinical Global Impression-Severity (CGI-S).

## Methods

### Procedure

**Figure 1:**
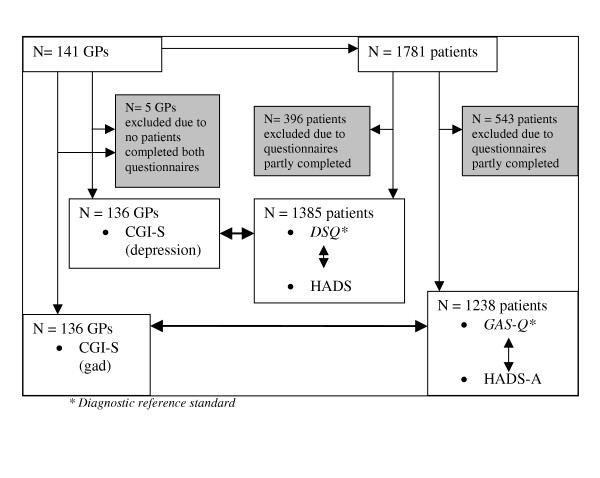
Flow chart study design.

The study is based on a cross-sectional study of GP's and their patients carried out in Germany, Scandinavia, and Finland [8,21]. A flow-chart over the study design is shown in Figure 1. Essential features of the design were: 1) Sampling of GPs geographically spread in Norway; 2) During three consecutive days in September 2001 all the GPs' patients were invited to take part in a study rating themselves on the three questionnaires concerning anxiety and depression;. 3) Blind to the patients' ratings, the GPs filled in the CGI-S in order to rate the clinical severity of eventual GAD and MDE in their patients. Exclusion criteria for patients were: age < 16 years, language difficulties, patients who required help to complete the questionnaires, and patients who came for prescription only, or for an accident/emergency.

### Sampling of GPs

The GPs in various parts of Norway were recruited as a convenience sample among those registered in the database of Wyeth Norway Ltd. The procedural information to the GPs was given in writing, and no special training of them for the study was undertaken. Among 141 participating GPs, 136 were eligible and 133 gave demographic data. Ninety GPs (68%) were men and 43 (32%) women. They had been working in primary care for a mean of 15 (SD 7) and 11 (SD 7) years, respectively, and 118 (89%) of them worked in group practice. The GPs consulted with a mean of 21.1 (SD 5.1) patients on an average day. There were no significant differences between genders of GPs with regard to number of consultations.

### Sampling of patients

Among the 1,781 patients included in the study, 1,385 (78 %) had valid ratings on the DSQ and the HADS-D, and 1,238 (70 %) on the GAS-Q and the HADS-A. The proportion of women in the two samples was 64% and 63%, respectively, with a mean age of 45 (SD 17) for women and 50 (SD 17) years for men. Further demographic characteristics of patients are shown in Table 1. The non-complying patients did not differ significantly from the compliers as to age and gender, which were the only data at disposal for attrition analyses.

**Table 1:** Sample characteristics.

**Variables**	**DSQ/HADS sample N = 1,385**	**GAS-Q/HADS sample N = 1,238**
Age, mean (SD):		
Female	45.7 (17.3)	45.2 (17.1)
Male	49.8 (17.3)	50.0 (17.7)

	**N (%)**	**N (%)**

Gender:		
Female	886 (64.0)	783 (63.2)
Male	499 (36.0)	455 (36.8)

Civil status:		
Married /paired relationship	934 (68.0)	848 (68.9)
Living alone	440 (32.0)	383 (31.1)

On sick leave:		
Yes	248 (20.2)	208 (18.9)
No	980 (79.8)	893 (81.1)

Prevalence rates:		
DSQ / GAS-Q positive	125 (9.0)	73 (5.9)
HADS-D / HADS-A (≥ 8)	256 (18.5)	357 (28.8)
CGI-S (dep / gad, ≥ 3)	337 (24.3)	217 (17.5)

### Diagnostic criteria and instruments

Psychiatric classification systems like DSM-IV and ICD-10 are based on the presence or absence of various operationalized diagnostic criteria. When structured interviews are used, the patients are asked for the presence of the diagnostic criteria by an interviewer. In contrast, in this study the patients rate themselves the diagnostic criteria for GAD (DSM-IV) on the Generalized Anxiety Questionnaire (GAS-Q) and for MDE (DSM-IV) on the Depression Screening Questionnaire (DSQ), and these patient ratings are used as diagnostic reference standard in this study.

The GAS-Q is a modification of the Anxiety Screening Questionnaire [22], and is a self-rating questionnaire developed to diagnose GAD according to DSM-IV and ICD-10. The GAS-Q consists of 20 items covering the diagnostic criteria for GAD in the DSM-IV. Test-retest reliability of the GAS-Q over a two-day retest period showed a kappa value of 0.74 for the diagnosis of GAD. Congruent validity comparing GAS-Q diagnosis with the DSM IV algorithm for GAD of the Composite International Diagnostic Interview showed a kappa of 0.72 [23].

The DSQ was made for patient-rating of MDE according to DSM-IV and ICD-10 [24] and was chosen as our reference standard. The DSQ is an 11 item questionnaire in which diagnostic criteria are rated on a three point scale, supplemented by three questions to assess the age at first and current episode, and the number of episodes according to the criterion A of MDE in DSM-IV. Consistent with the DSM-IV criteria, a diagnosis of MDE was assigned when at least five of the items were rated as positive by the patient. In the German part of the European study, the internal consistency of the DSQ showed a Cronbach's coefficient alpha of 0.83 [25]. Test-retest reliability over a two-day period found a kappa value of 0.82 for MDE [8]. Tests of the DSQ diagnosis versus diagnosis of MDE based on structured interview showed a kappa 0.89 [26].

The HADS consists of seven items for anxiety (HADS-A) and seven for depression (HADS-D). The items are scored on a four-point scale from zero (not present) to three (considerable). The item scores are added, giving sub-scale scores on the HADS-A and the HADS-D from zero to 21. In this study valid HADS subscale scores were defined as having answered at least five of seven items on both the HADS-A and the HADS-D. In order to be valid in patients with somatic problems, the HADS items were based on the psychological aspects of anxiety and depression. The anxiety items were concentrated on general anxiety, and five of the items were close to the diagnostic criteria of GAD. The depression items were based on anhedonia, which is considered to be one of the essential criteria of depression [27]. The concurrent validity of the HADS compared to other questionnaires for anxiety and depression is described between 0.60 and 0.80 for both sub-scales [13].

The CGI-S is a standardized assessment tool that is widely used as an outcome measure in research [28]. The CGI-S had the following wording: "In your clinical judgement how severely does this patient suffer from MDE/GAD?" The ratings of CGI-S were: 1 = not ill at all, 2 = a borderline case, 3 = only mildly ill, 4 = moderately ill, 5 = seriously ill and 6 = extremely seriously ill. The CGI-S scale was dichotomised into 1–2 = not ill, 3–6 = ill, but we also explored the frequency of cases by a CGI-S score of ≥ 2 (= borderline case).

### Statistical methods

The statistical analyses were carried out with the SPSS for Windows, version 11.0. Principal Component Analysis (PCA) with oblique rotation was performed to explore the factor structure of the HADS. Internal consistency of the HADS-A and the HADS-D was tested using Cronbach's coefficient alpha. Pearson's correlation coefficient was used for estimation of the overlap between the subscales. Sensitivity and specificity were calculated for different cut-off values for the HADS-A, the HADS-D, and the CGI-S in relation to the prevalence rate of GAD identified with GAS-Q and the rate of MDE identified with DSQ. Sensitivities and specificities by optimal cut-off were used to calculate the rates of true and false positive and negative cases. The Receiver Operating Characteristics (ROC-curve) were depicted graphically, and the Area Under the Curve (AUC) were calculated for the HADS-A, the HADS-D and the CGI-S against the GAS-Q and the DSQ as reference standards. The associations of age and gender to caseness on the instruments were examined by logistic regression analyses. All significance tests were two-tailed, and p-values < .05 were reported as significant.

### Ethics

The Committee for Medical Ethics of Health Region East of Norway approved this study. The participants delivered informed consent after written information about the study. Wyeth Norway Ltd paid the GPs a fixed sum of EUR 15 per patient in addition to their normal salary. No employees of Wyeth Ltd. were present in any of the general practices during the day of inclusion. The national study leader coordinated the study, and Wyeth Norway Ltd functioned as sponsor of the study. This implied that employees of Wyeth Norway Ltd brought the material for the study to the GPs and later on collected the forms, but otherwise had no active part in the study. The company made no use of the collected data or analyses in their marketing. The study leader and his co-authors had no restrictions as to the content of the publications from Wyeth Norway Ltd, and the company did not want to review any manuscripts before submission.

## Results

### Prevalence rates

According to the DSQ, 9.0% (CI 7.6 – 10.7%) of patients had MDE, and based on the GAS-Q 5.9% (CI 4.7 – 7.4%) had GAD. Prevalence rates for HAD-D (≥8) and HADS-A (≥8) were 18.5% (CI 16.5 – 20.6%) and 28.8% (CI 26.3 – 31.5%), respectively. According to GPs' clinical judgement by CGI-S (≥ 3) the prevalence rates were 24.3% (CI 22.1 – 26.7%) for MDE and 17.5% (CI 15.5 – 20.0%) for GAD. The associations between female gender and CGI-S caseness of depression (OR 1.5, p = 0.004, CI 1.3–1.9) and HADS-A caseness of anxiety disorder (OR 1.4, p = 0.013, CI 1.1 – 1.8) were both significant. Age significantly reduced the prevalence of caseness with 1–3 % on DSQ, GAS-Q and HADS-A. Based on a GPs' CGI-S score cut-off ≥ 2, the prevalence rates were 38% for MDE and 25% for GAD.

### Psychometrics of the HADS

The internal consistency of the HADS-A and the HADS-D showed coefficient alpha of 0.89 and 0.86, respectively. PCA with varimax rotation of all 14 HADS items, extracted two factors both with Eigen-value of 4.13, and that factor solution comprised 59% of the explained variance. Anxiety and depression items loaded on separate factors. The anxiety and depression sub-scales shared 54% of the explained variance.

### Case-finding abilities of the HADS

**Figure 2:**
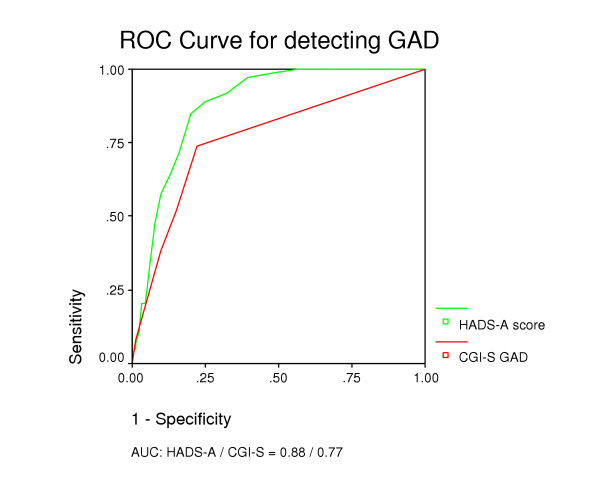
Receiver Operating Curves for HADS-A and CGI-S for detecting GAD.

**Figure 3:**
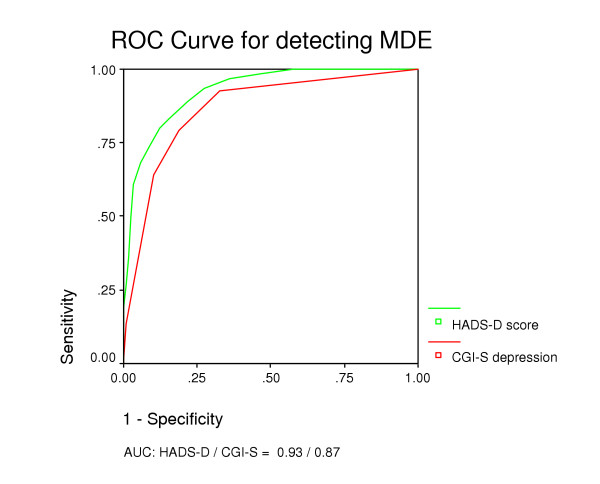
Receiver Operating Curves for HADS-D and CGI-S for detecting MDE.

Case-finding abilities for various cut-off values of the HADS-A and the HADS-D are shown in Table 2. By cut-off score of ≥ 8 on HADS-A, GAD was detected with a sensitivity of 0.89 and a specificity of 0.75. Using the same cut-off for the HADS-D, MDE cases were detected with a sensitivity of 0.80 and a specificity of 0.88. Identification of GAD showed an AUC of 0.88 for the HADS-A (Figure 2). For identification of MDE, the AUC was 0.93 for HADS-D (Figure 3).

**Table 2:** Sensitivity and specificity for HADS-A/D and CGI-S.

		**Generalized Anxiety Disorder ** (N = 1,238)			**Major Depressive Episode ** (N = 1,385)		
	**Score**	**N (%)**	**Sensitivity**	**Specificity**	**N (%)**	**Sensitivity**	**Specificity**

**HADS – A/D**	**5**	637 (51.5)	0.99	0.52	463 (33.4)	0.94	0.73
	**6**	528 (42.6)	0.97	0.61	384 (27.7)	0.89	0.78
	**7**	444 (35.9)	0.92	0.68	301 (21.7)	0.83	0.84
	**8**	357 (28.8)	0.89	0.75	256 (18.5)	0.80	0.88
	**9**	296 (23.9)	0.85	0.80	201 (14.4)	0.74	0.91
	**10**	236 (19.1)	0.71	0.84	156 (11.3)	0.68	0.94
	**11**	198 (16.0)	0.64	0.87	118 (8.5)	0.61	0.97
	**12**	157 (12.7)	0.58	0.90	91 (6.6)	0.50	0.98
	**13**	127 (10.3)	0.47	0.92	64 (4.6)	0.36	0.99
	**14**	95 (7.7)	0.34	0.94	41 (3.0)	0.26	0.99

							

**CGI-S**	**2**	314 (25.4)	0.74	0.78	529 (38.2)	0.93	0.63
	**3**	217 (17.5)	0.52	0.85	337 (24.3)	0.79	0.81
	**4**	141 (11.4)	0.38	0.90	208 (15.0)	0.64	0.90
	**5**	20 (1.6)	0.08	0.99	29 (2.1)	0.14	0.99
	**6**	2 (0.2)	0.00	1.00	2 (0.1)	0.02	1.00

### Comparison of GP-rated and patient-rated case identification

Using the GPs' CGI-S score of ≥ 3 as cut-off for a positive diagnosis, GAD was detected with a sensitivity of 0.52 and a specificity of 0.85 (Table 2). MDE was detected with a sensitivity of 0.79 and specificity of 0.81 by the same CGI-S cut-off level. Identification of GAD and MDE with the CGI-S showed AUCs of 0.77 (Figure 2) and 0.87 (Figure 3), respectively.

Based on the sensitivities and specificities of the HADS-A by cut-off ≥ 8 and the CGI-S by cut-off ≥ 3 (Table 3) we found that the true positive disorder rate for the HADS-A (5.3%) was close to reference standard (5.9%) and significantly better than for the CGI-S (3.1%). The opposite was found for the rates of true positive healthy cases. The total true hit rate of GAD and non-GAD was significantly better for the GP-rated CGI-S (83%) than for the patient-rated HADS-A (76%).

**Table 3:** Classification of patients with eventual GAD and MDE.

	**Generalized Anxiety Disorder (GAS-Q Positive 73/1,238 = 5.9%)**		**Major Depressive Episode (DSQ Positive 125/1,385 = 9.0%)**	
	**Patients' HADS-A ≥ 8**	**GPs' CGI-S ≥ 3**	**Patients' HADS-D ≥ 8**	**GPs' CGI-S ≥ 3**
**Sensitivity / Specificity**	**0.89 / 0.75**	**0.52 / 0.85**	**0.80 / 0.88**	**0.79 / 0.81**

**Classification**	**% (95% CI)**	**% (95% CI)**	**% (95% CI)**	**% (95% CI)**
True positive disorder	5.3 (4.1–6.5)*	3.1 (2.1–4.1)	7.2 (5.8–8.6)	7.1 (5.7–8.5)
False positive disorder	23.5 (21.1–25.9)*	14.1 (12.2–16.0)	10.9 (9.3–12.5)	17.3 (15.3–19.3)*
Observed disordered	28.8 (26.3–31.3)*	17.2 (15.1–19.3)	18.1 (16.1–20.1)	24.4 (22.1–26.7)*
True positive healthy	70.6 (68.1–73.1)	80.0 (77.8–82.2)*	80.1 (78.0–82.2)*	73.7 (71.4–76.0)
False positive healthy	0.6 (0.2–1.0)	2.8 (1.9–3.7)*	1.8 (1.1–2.5)	1.9 (1.2–2.6)
Observed healthy	71.2 (68.7–73.7)	82.8 (80.7–84.9)*	81.9 (79.9–83.9)*	75.6 (73.3–77.9)
Total rightly classified	75.9 (73.5–78.3)	83.1 (81.0–85.2)*	87.3 (85.5–89.1)*	80.8 (78.7–82.9)
Total wrongly classified	24.1 (21.7–26.5)*	16.9 (14.8–19.0)	12.7 (10.9–14.5)	19.2 (17.1–21.3)*

*The difference shows a statistical significance of p ≤ 0.05

For MDE no significant difference was observed between rates of true positive disorder (Table 3). For true non-depression rate, the HADS-D (80%) showed a significantly better hit rate than the CGI-S (74%). The proportion of totally right classified depressed patients was significantly better for the patient-rated HADS-D (87%) than for the GP-rated CGI-S (81%).

## Discussion

### Strengths and limitations

Compared to former studies from general practice the high number of patients and GPs in our study is a strength due to increased variance and reduced biases. The big sample sizes and a responder-rate above 70% among patients give adequate statistical power to the performed analyses. Our sample consists of geographically spread GPs who's working experience and gender distribution is representative for GPs in Norway [29]. Patients' age and gender is representative for patients attending GPs in Scandinavia [30]. We also consider as a strength that the GPs were blind to the HADS scores of the patients when they made their diagnostic evaluations.

It is a weakness of our study that we did not employ structured interviews for the establishment of reference standard diagnoses of GAD and MDE. However, the reference standards used by us comprise the same diagnostic criteria, are well described, and have shown good validity in relation to structured interviews [23,25]. When both the HADS and the reference standards are self-rating instruments, the HADS might be systematically biased with falsely high sensitivity and/or specificity in relation to the reference standard. On the other hand, an interview could introduce observer bias in the interpretation of symptoms, which is eliminated using self-ratings. The reference standard questionnaires used in our study gave prevalence rates for GAD and MDE in general practice that were in accordance with the prevalence rates reported by Üstün & Sartorius [1], and this added some validity to our approach. Our design did not take into account the GPs' knowledge about the patients' somatic symptoms or psychosocial situation, which could be relevant information for the GPs in their diagnostic considerations. However, studies have shown that in non-clinical samples chronic somatic problems [16] and demographic variations [31] have only modest influence on the HADS scores.

The use of the CGI-S as a diagnostic instrument could be discussed since the instrument only evaluates the severity of the case. Severity is not a clear concept, and it is implicit in such ratings that the GPs are familiar with both mild and severe cases of GAD and MDE, although that hardly is the case. Further, the GPs could be biased in direction of false positive diagnoses since they took part in a sponsored study concerning these mental disorders.

### Comparison with existing literature

The internal consistency of the HADS was found in accordance with other studies [16,13]. The replication of the original two-factor structure of the HADS among primary care attenders has been discussed. A Dutch validation study [32] found evidence for the original two-factor structure among a sample (n = 112) of consecutive general practice patients. Data from a large non-clinical population give support to a s two-factor-structure of the HADS [16] in sub-samples with higher mental symptom levels than in the general population. In our sample from general practice, the HADS showed good separation of items, moderate inter-correlation, and a distinct two-factor structure. These results support the robustness of the HADS as a psychometrically adequate self-rating instrument for patients attending general practice.

An optimal balance between sensitivity and specificity is requested of a good questionnaire. From a clinical perspective high sensitivity might be seen the most important concern for a screening instrument, giving minimal number of false negative cases at the sacrifice of some false positive cases.

In general we found that the patient-rated HADS-A/D had better diagnostic ability than CGI-S rated by GPs (Figure 2 and 3) in relation to GAD and MDE. However, taking into regard the prevalence rate of 5.9% of GAD in general practice, the GPs' ability to recognise people not suffering from GAD (Table 3) is significant superior to that of the HADS-A and important in the clinical setting. With a prevalence rate of 9% for MDE in general practice the total proportion of patients correctly identified by HADS-D was significantly higher than that of GPs using the CGI-S.

### Implications for future research or clinical practice

HADS showed satisfying psychometric properties in the general practice setting, which is of importance for future research. We found that GPs mainly recognized GAD by exclusion and MDE by inclusion, but still they had a considerable proportion of misclassifications. GPs' diagnostic precision in clinical practice is improved by supplementing HADS scores. The advantage of HADS is its feasibility of completion and well-established cut-off scores for clinically relevant caseness.

## Conclusion

The psychometrics of the HADS was found to be excellent in this sample from general practice. The recommended cut-off score for caseness on the HADS-A and the HADS-D of ≥8 seemed appropriate for detecting GAD and MDE among patients attending primary care. In regard to prevalence rates, the GPs should positively trust their sensitivity in diagnosing MDE, and their specificity in diagnosing GAD by exclusion of patients without anxiety. Patient-rated HADS could represent a useful supplement to GPs' own clinical judgment.

## Competing interests

Wyeth Norway Ltd may gain financially from the publication of this manuscript. The manuscript concerns the prevalence and under identification of GAD in primary care, for which Wyeth Norway Ltd. sells venlafaxine (Efexor^®^). We have no financial interest to declare.

## Authors' contributions

The authors have carried out the study together. IO organised and analysed data and wrote the first draft of the article. AM contributed to the analyses and the interpretation of the data and constructed the graphs. AAD made contributions to conception and design and revised the manuscript critically.

## Pre-publication history

The pre-publication history for this paper can be accessed here:


